# War‐Induced Behavioral Change in Spotted Hyena (*Crocuta crocuta*) Populations in Tigray, Northern Ethiopia

**DOI:** 10.1002/ece3.70920

**Published:** 2025-02-24

**Authors:** Gidey Yirga, Robert P. Freckleton, Andrew P. Beckerman

**Affiliations:** ^1^ Department of Biology Mekelle University Mekelle Tigray Ethiopia; ^2^ School of Biosciences University of Sheffield Sheffield UK

**Keywords:** Battle, depredation, diet, hyena, scavenging

## Abstract

In Tigray, northern Ethiopia, there is a long tradition of peaceful coexistence between spotted hyenas ( *Crocuta crocuta* ) and humans. While historically the coexistence has been relatively stable, we assessed the impact of the recent war in Tigray on this coexistence. We investigated the effects of war on the scavenging and hunting behavior of spotted hyenas, and the consequences for local people in Tigray. We compared current spotted hyena foraging at six battle sites with six control sites across Tigray using diet analysis, hyena abundance through playback experiments, and assessed human−wildlife interactions via semistructured interviews in 1200 households. Spotted hyena diets at both site types consisted exclusively of domestically derived prey; however, the composition of prey species differed significantly (*χ* ^2^ = 64.03, df = 6, *p* = 0.001). Human hair was prevalent in hyena scats from battle sites but was absent in scats collected from the control sites. In total, 318 hyenas responded to 48 call stations, response rates were significantly higher at battle sites ($\bar{x}$ = 36.7 ± 9.7 SD) than at control sites ($\bar{x}$ = 16.3 ± 14.6 SD). There were several lines of evidence that human−wildlife interactions were more negative. Reported livestock predation was 18.85% higher at battle sites, with 78.5% of depredation events occurring during the war, compared to 6% pre‐war and 15.5% post‐war. We conclude that changes in hyena feeding behavior during the war and siege period can be linked to changes in the availability of scavengable food sources. These results yield insight not only into the consequences of war for the people of Tigray but also into how the many armed conflicts in regions with large scavenger/carnivore populations may have long‐lasting impacts on human−wildlife conflict around the globe.

## Graphical abstract

Spotted hyena diet and habitat shifts are driven by war and conflict in Ethiopia, changes specifically linked to the availability of human casualties of war. We found that the spotted hyena scats contained primarily domestic livestock at both the battle and control sites. Human hair was prevalent in hyena scats from battle sites but was absent in scats collected from the control sites.

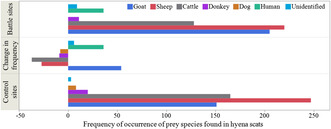

## 1 Introduction

Over the past 60 years, war has occurred in at least two thirds of the world's biodiversity hotspots (Hanson et al. 2009). Wars, widely distributed in Africa with frequent outbreaks in sub‐Saharan Africa, have detrimental effects on wildlife habitats and populations (Dudley, Ginsberg, and Plumptre 2002; Shambaugh, Oglethorpe, and Ham 2001). Despite widespread and recurrent war, little ecological detail is available on the consequences of war (Gaynor et al. 2016).

War has profound ecological and behavioral impacts on wildlife populations. Large‐mammal population declines during wartime often result from a combination of subsistence hunting and commercial wildlife trafficking. For instance, during the war in Africa between 1946 and 2010, elephants ( *Loxodonta africana* ), hippos ( *Hippopotamus amphibius* ), giraffes ( *Giraffa camelopardalis* ), and other large mammals were hunted by combatants and civilians for meat and ivory (Daskin and Pringle 2018). While large carnivores such as spotted hyenas ( *Crocuta crocuta* ) were rarely hunted for food, their populations declined significantly due to the depletion of prey. Behavioral changes in large carnivores are also evident during war times. For example, during the Mozambican Civil War (1977–1992), lions ( *Panthera leo* ) became more nocturnal to avoid human activity (Stalmans et al. 2019). Similarly, Tigers increasingly preyed on humans in the Sundarbans during and after World War II (Chakrabarti 2009), demonstrating how war‐driven disruptions can alter predator behavior.

In a remarkable case of coexistence between humans and wildlife, we have previously reported that large spotted hyena populations live alongside human communities in Tigray, Ethiopia, without coming into conflict (Yirga et al. 2013). Hyenas in Tigray are specialized in waste consumption: they benefit from waste that people dispose of, and residents benefit from hyena waste‐clearing services (Yirga et al. 2013). While historically the coexistence has been relatively stable, we investigate the impact of the recent war in Tigray on this coexistence.

The war in Tigray is widely regarded as one of the most tragic and deadly conflicts of the 21st century (Naranjo 2023). This large‐scale war involved the Ethiopian National Defense Forces (ENDF) and their allies, including the Amhara Special Forces, Amhara militia, Ethiopian Federal Police, Fano (an informal armed group in the Amhara region), Afar Special Forces, and the Eritrean Defense Forces (EDF) fighting against the Tigray Defense Forces (TDF) from November 2020 to November 2022. The war resulted in an estimated 383,000–600,000 civilian deaths and 250,000–600,000 combatant fatalities (Hjelmgaard et al. 2022). Furthermore, the war caused severe humanitarian and ecological devastation. Humanitarian aid was systematically blocked from entering Tigray, while troops deliberately destroyed essential crops. This destruction, compounded by locust infestations, left the Tigray region socially and ecologically devastated by the war. Before the war, hyenas in Tigray concentrated in urban areas around waste dumps and anthropogenic food was the main source of food for hyenas, which in this region typically scavenge on bones, skin, food waste, and carcasses of livestock that have died from disease and drought (Gade 2006; Abay et al. 2011; Yirga et al. 2012; Baynes‐Rock 2013). This points to hyena provision of critical ecosystem and civic service tied to waste disposal and even carbon sequestration (Yirga et al. 2024). Our previous work showed a positive relationship between hyenas and human concentrations, even at high densities in this region, hyenas eat almost exclusively anthropogenic food (Yirga et al. 2017).

A consequence of the war and associated devastation was that civic waste removal ceased due to unrest and fuel shortages. This not only impacted the human population but also deprived urban hyenas of their main food source. During the war, a complete military siege left people starving, significantly reducing the availability of anthropogenic food sources for hyenas, particularly in urban areas. However, the war created a ghastly yet unique opportunity for hyenas to scavenge the remains of unarmed civilians and combatants.

Given the changes in resource availability, we hypothesized that the war in Tigray has likely had a strong effect on the scavenging behavior and diet of hyenas. Here, we focus on documenting the pattern and mechanism driving these changes. First, we evaluated the hypothesis that the human remains became part of the diet of hyenas with impact on the relative abundance of pre‐war diet items. Second, we evaluate the hypothesis that density increased in rural and battlefield areas, associated with this diet change. Finally, we document the associated changes in human−wildlife interaction in both urban and rural/battlefield locations associated with these diet and abundance changes.

## 2 Study Area

Tigray is a historic region in Ethiopia, home to a population of approximately eight million people. It is located between 36°26′45″ E—39°59′28″ E longitude and 12°15′20″ N—14°50′44″ N latitude in the northeastern part of the country. The region features diverse topography, with elevations ranging from 500 to 3954 m above sea level, and spans an area of about 53,000 km^2^. Annual mean temperatures vary from 12°C to 37°C, while annual rainfall ranges from around 200 mm in the lowlands to over 1000 mm in the highlands (Sara 2010). Agriculture is the primary driver of the region's economy.

Tigray is located within 50 km of the Red Sea region (the Horn of Africa), an area with a long history of geopolitical instability and social turmoil (Plaut and Vaughan 2023; Waal 2015; Plaut 2013). The region lies within the African drylands, specifically the Sudano‐Sahelian zone. Recurrent severe droughts, driven by climate change and extreme weather variability, affect the region every 2–3 years. Tigray has long experienced extreme land degradation and deforestation. Some vegetation remains around public sites and churches, mainly consisting of *Acacia* spp. and *Eucalyptus* spp. Wild prey is chronically depleted, and predators, including hyenas and African wolves ( *Canis lupus* lupaster)—hereafter referred to as wolves—rely primarily on anthropogenic food sources (Yirga et al. 2012, 2013, 2015). Hyenas and wolves are commonly seen roaming the city at night, while during the day, they hide in the surrounding agropastoral landscape.

**FIGURE 1:**
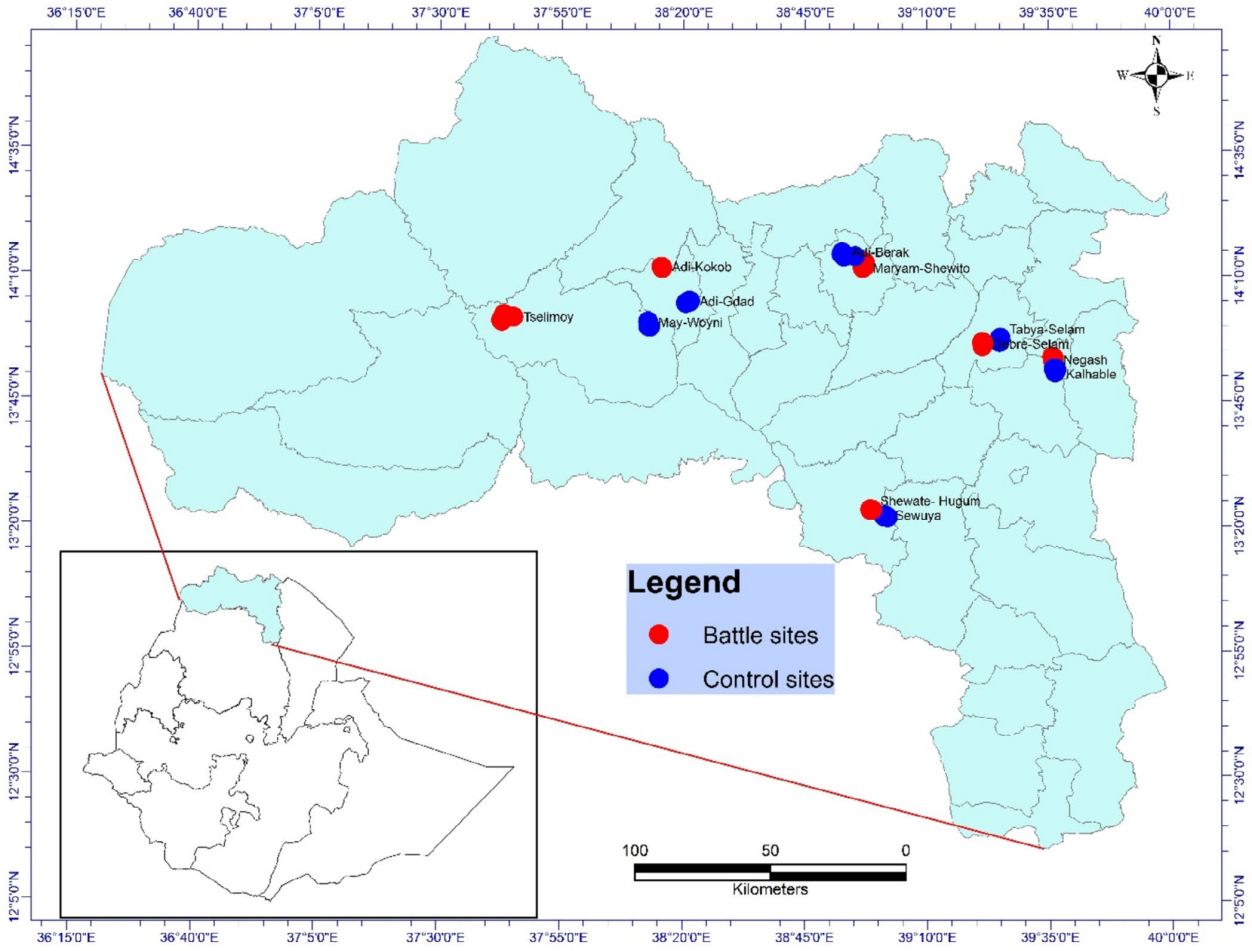
Map of Ethiopia showing the location of Tigray and map of Tigray showing the location of the study sites categorized as battle sites and control sites.

The research was carried out from November 2023 to March 2024 in Tigray, northern Ethiopia (Figure 1). We established 12 study sites in two different types of locations: Battle sites (*n* = 6) where heavy fighting involving tanks, artillery, machine guns, fighters, and bombers including drones took place, and “control sites” (*n* = 6) where no fighting took place. The battle sites consisted of Tselimoy, Adi‐Kokob, Maryam‐Shewito, Debre‐Selam, Negash, and Shewate‐Hugum, and the control sites consisted of May‐Woyni, Adi‐Gdad, Adi‐Berak, Tabya‐Selam, Kelhable, and Sewuya. All study sites are severely degraded, human‐dominated, and natural prey‐depleted areas. As Figure 1 indicates, our design provides six paired battle‐control replicates. The study sites were selected with the assistance of local residents and administrative officials to ensure that the locations were both accessible and relevant to the research objectives. Each pair of battle and control sites was located within the same district and similar geographical areas. The sites were comparable in terms of altitude, activity patterns, livestock ownership, and ecological conditions.

## 3 Methods

### 3.1 Dietary Analysis

We evaluated differences in the diet and relative abundance of items between the control and battle sites. A total of 603 hyena scats were collected from battle sites: Tselimoy (*n* = 101), Adi‐Kokob (*n* = 100), Maryam‐Shewito (*n* = 100), Debre‐Selam (*n* = 100), Negash (*n* = 100), and Shewate‐Hugum (*n* = 101). Similarly, a total of 600 hyena scats were collected from control sites: May‐Woyni (*n* = 100), Adi‐Gdad (*n* = 100), Adi‐Berak (*n* = 100), Tabya‐Selam (*n* = 100), Kelhable (*n* = 100), and Sewuya (*n* = 100). We collected scat samples in plastic bags while walking transects around the villages. We extracted hair from scat and compared these with hair in our reference guide (Beveridge and Hoogen 2013) to establish the composition of the prey species. We prepared human hair as a reference since it was not in the reference guide. Hairs were analyzed in scale patterns using a microscope (stereo dissecting microscope) at 10× magnification.

We assessed whether diets were nonrandom (e.g., hyenas feed with choice rather than selecting items in proportion to their availability). We tested for differences in prey species composition between battle and control sites using a chi‐square test of independence. We visualized raw frequencies for each type of site and the difference in frequencies.

### 3.2 Assessment of Abundance

We evaluated differences in abundance between the two types of sites using vocalization playback methods. We measured the abundance of hyenas using playback experiments at battle and control sites between 18:00 and 24:00, as previously done in Africa (Mills, Juritz, and Zuccini 2001; Bauer 2007; Yirga et al. 2015). We played hyena whooping and laughing sounds for 1 h on an MP3 player connected to a megaphone (Monacor 45) placed on top of a vehicle. Each experiment consisted of two rounds of 20 min broadcast and 10 min silence, and the speaker was rotated 90° every 5 min. We counted responding hyenas in the dark based on eye reflections from a powerful torch (TQC inspector flashlight). To minimize the probability of duplicate counts, the calling stations were at least 6 km apart, and the neighboring stations were sampled consecutively on the same day. The calling stations were located along the roads in open areas to allow visibility and GPS coordinates of the locations were recorded. First, we do a nonparametric van der Waeden test to test differences in response rates between the two site types by using JMP‐17 software.

### 3.3 Human−Wildlife Conflict

We evaluated changes in human−wildlife interactions via semistructured interviews in 1200 systematically selected households in battle and control sites, with 100 households sampled from each study site. The survey collected information on livestock predation events and the existence of direct human attacks by predators across three distinct periods: before, during, and after the war (November 2018 to March 2024). Interviews with household heads were conducted by trained assistants in the local language (Tigrinya) and only after obtaining informed consent and explaining the purpose of the study to each participant.

For systematic sampling, every third household was selected in each study site. We recorded conditions of human attack (age, sex, year, and location), livestock predation events (species, number, year, and location), and responsible predators. We also estimated from these data the number of livestock attacks caused by predators (hyenas, leopards ( *Panthera pardus* ), and wolves) at our study sites in different time periods, and the data reported are by the sample of respondents. We note that there were no attacks on or feeding off live human beings. We subsequently tested for differences in livestock predation by hyenas, wolves, and leopards in battle and control sites during pre‐war, war, and post‐war periods with a nonparametric van der Waeden test using JMP‐17 Software.

### 3.4 Ethics Statement

This study was conducted with the approval of Mekelle University and local administration officials. The purpose of the study was clearly explained to all participants before their involvement. Household interviews were conducted only after obtaining informed consent from each participant, ensuring their voluntary participation. Ethical considerations, including confidentiality and respect for the participants' autonomy, were strictly adhered to throughout the study.

## 4 Results

### 4.1 Hyena Diet in Battle and Control Sites

**FIGURE 2:**
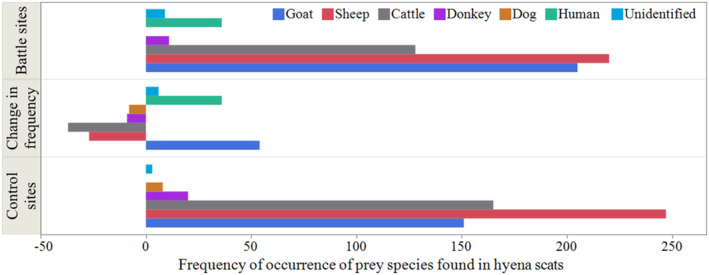
Frequency of occurrence of prey species found in hyena scats in battle and control sites and change in frequency of occurrence of prey species (battle‐control), based on analysis of 1203 scats. *The reference hair also included natural prey species, but these did not match the unidentified hairs.

A total of 1203 hyena scats were analyzed. The hyena diet consisted of exclusively domestically derived prey items at both battle and control sites (Figure 2). Overall, the diets were not random with a distribution consisting of sheep (38.8%, *n* = 467), goat (29.6%, *n* = 356), cattle (24.3%, *n* = 293), human (3%, *n* = 36), donkey (2.6%, *n* = 31), dog (0.7%, *n* = 8), and unidentified (1%, *n* = 12). Notably, we found human hair in hyena scats from battle sites (6%), but we did not find human hair in scats collected from control sites. There was a highly significant difference (*χ* ^2^ = 64.03, df = 6, *p* = 0.001) in prey species composition between battle and control sites. Our analysis of diet change indicates that human remains and goats showed greater frequency of occurrence in battle sites than in control, dominated by moving away from dogs and donkeys (Figure 2).

### 4.2 Hyena Abundance in Battle and Control Sites

**FIGURE 3:**
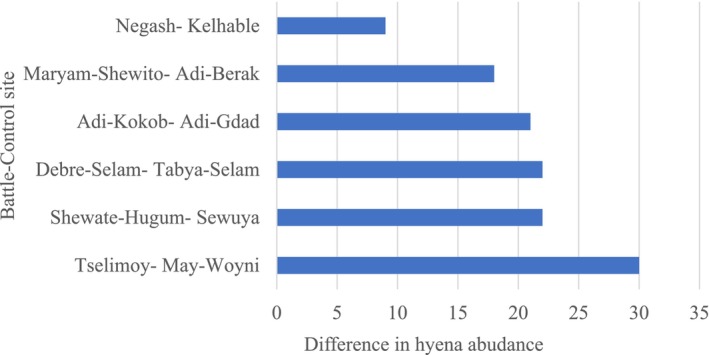
Increased frequency of hyena abundance among all site pairs in Tigray, northern Ethiopia. The same number of calling stations for each site (four per site) and the bar represents the difference in frequency of hyena abundance (battle control) sites.

We detected more hyenas on battlefield sites than on control sites (Figure 3). In total, 318 hyenas responded to 48 call stations among the six battle and six control sites in Tigray. Of the 24 calling stations at the battle sites, a total of 220 hyenas responded. On the contrary, only 98 hyenas responded to the 24 calling stations in control sites (Table 1).

**TABLE 1:** The number of spotted hyenas that responded to each calling station at battle and control sites in Tigray, northern Ethiopia.

Site type	Location	Calling station 1	Calling station 2	Calling station 3	Calling station 4	Total
Battle sites	Tselimoy	30	0	0	0	30
	Adi‐Kokob	0	4	20	6	30
	Maryam‐Shewito	0	0	0	50	50
	Debre‐Selam	0	25	9	8	42
	Negash	2	10	15	16	43
	Shewate‐Hugum	0	0	10	15	25
Control sites	May‐Woyni	0	0	0	0	0
	Adi‐Gdad	4	2	1	1	8
	Adi‐Berak	6	0	8	18	32
	Tabya‐Selam	3	3	11	16	33
	Kelhable	0	10	2	10	22
	Sewuya	0	0	0	3	3

Response rates were significantly higher at battle sites ($\bar{x}$ = 36.7 ± 9.7 SD) than at control sites ($\bar{x}$ = 16.3 ± 14.6 SD) (*χ* ^2^ = 47, df = 1, *p* = 0.001; Figure 3). Figure 3 visualizes the variation between the paired sites and shows the uniform increase in abundance at the battle sites.

## 5 Survey Results

### 5.1 Human Attacks and Human Body Resources

The survey results did not indicate direct human attacks by hyenas during the war period. However, respondents reported that hyenas scavenged on the remains of unarmed civilians killed by coalition troops at the study sites (Table 2). This behavior was observed primarily due to the lack of timely burial, as respondents indicated that they were prohibited from burying the bodies, leaving them exposed for days.

**TABLE 2:** Number of respondents reporting spotted hyenas scavenging on the remains of unarmed civilians killed by coalition troops at study sites in the Tigray region, northern Ethiopia.

Site type	Location	Number of respondents witnessing hyena scavenging
Battle sites	Maryam‐Shewito	100
	Negash	16
	Debre‐Selam	11
	Adi‐Kokob	8
	Tselimoy	3
Control sites	Adi‐Berak	6
	Adi‐Gdad	5
	Kelhable	4

The availability of human corpses as a food resource for hyenas was higher at battle sites compared to control sites. Battle sites, total: 175 unarmed civilians (aged 8–76 mostly males) were killed; Maryam‐Shewito (*n* = 140), Negash (*n* = 14), Debre‐Selam (*n* = 10), Adi‐Kokob (*n* = 8), and Tselimoy (*n* = 3). Control sites, total: 15 unarmed civilians; Adi‐Berak (*n* = 6), Adi‐Gdad (*n* = 5) and Kelhable (*n* = 4).

### 5.2 Livestock Predation at Battle and Control Sites

There were more reports of livestock predation in the battle than in the control sites. For example, at the battle sites, a total of 763 livestock were reported killed by hyenas, leopards, and wolves, consisting of goats (43.9%, *n* = 335), sheep (37.8%, *n* = 288), chicken (11%, *n* = 84), donkey (4.3%, *n* = 33), and cattle (3%, *n* = 23) in decreasing order. At the control sites, a total of 642 livestock were reported killed by hyenas, leopards, and wolves, consisting of sheep (35.5%, *n* = 228), goats (29.9%, *n* = 192), chicken (24%, *n* = 154), donkey (6%, *n* = 39), and cattle (4.5%, *n* = 29) in decreasing order.

Livestock predation was higher overall during the war. At battle sites, livestock predation was significantly higher during the war period (*χ* ^2^ = 39.55 df = 2, *p* = 0.0001); 78.5% (*n* = 599) of the depredation events occurred during the war period followed by 6% (*n* = 46) and 15.5% (*n* = 118) during the pre‐ and post‐war periods, respectively. During the war, livestock predation rates were 13 times higher compared to before the war, and predation rates were nearly three times higher (15.5%) even after the war ended compared to before (6%) the war at the battle sites. Similarly, at the control sites, livestock predation was significantly higher during the war period (*χ* ^2^ = 39.55 df = 2, *p* = 0.0001); 51.4% (*n* = 330) depredation occurred during the war period than during the pre‐war (16.4%, *n* = 105) and after‐war (32.2%, *n* = 207) periods. During the war the livestock predation rates were three times higher compared to before the war and were two times higher after the war (32.24%) compared to before the war (16.36%) at the control sites. Compared to before the war, livestock predation at control sites was significantly higher during the post‐war period (*χ* ^2^ = 5.59 df = 2, *p* = 0.0370).

Hyenas are the largest being responsible for the increase in the reported livestock predation, likely scavenging on human remains. At battle sites, predation by hyenas (51.3%, *n* = 391) was significantly higher compared to wolves (30.1%, *n* = 230) and leopards (18.6%, *n* = 142) (*χ* ^2^ = 39.55, df = 2, *p* = 0.0001) during the war period. Similarly, at the control sites, hyena predation of livestock (39.7%, *n* = 255) was significantly higher (*χ* ^2^ = 6.5916, df = 2, *p* = 0.0370) than by wolves (33.2%, *n* = 213) and leopards (27.1%, *n* = 177) during the war period.

**FIGURE 4:**
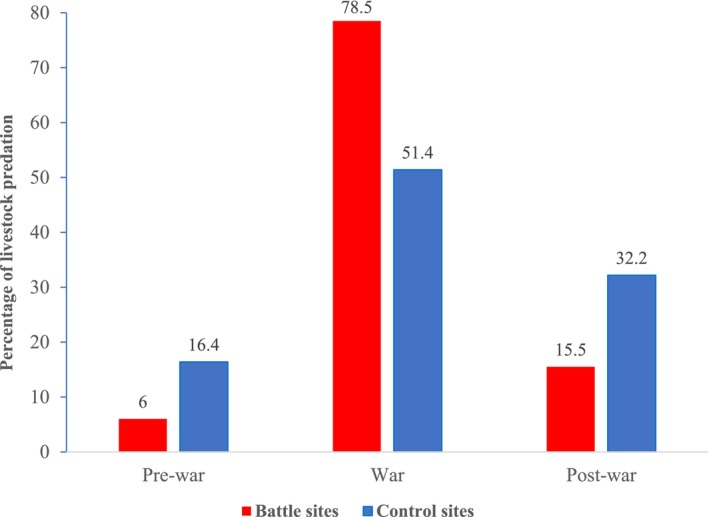
Percentage of livestock predation events at control versus battle sites—before, during, and after the war in Tigray, Ethiopia.

Reported livestock predation events by all predators increased during the war. Survey information revealed that predator identity included hyenas, leopards, and wolves, and prey included sheep, goats, chickens, donkeys, and cattle. The predation of cattle and donkeys in our study area was reported to be exclusively caused by hyenas. The frequency and severity of hyena, leopard, and wolf attacks on livestock significantly increased during the war period at both battle and control sites. Figure 4 shows the predator–prey identity specific shift in depredation associated with war.

The households of the battle sites experienced 18.85% higher livestock predation patterns compared to the households of the control sites. Donkey predation was substantially reduced during the war at the battle sites, as the donkeys went to battle with the combatants and carried weapons, water, food, and other military supplies on the front line.

## 6 Discussion

War has the potential to have an impact on large carnivores through many mechanisms. In this study, we have focused on the unique situation in Tigray, Ethiopia, where hyena have coexisted with humans for centuries but where prolonged war has created conditions in which scavenger diets, abundance, and propensity for human−wildlife conflict have changed because of the prevalence of human corpse remains driving a habitat and diet shift. We combined scat analysis, abundance estimation, and structured surveys in replicate battle‐control sites across Tigay to characterize these changes.

### 6.1 Hyena Diet in Battle and Control Sites

We found that the hyena scat contained more human hair at the battle sites, indicating a greater incidence of hyenas scavenging human carcasses. Human hairs in the scats were not likely to be from direct attacks on humans, as they were not reported during the study period. Instead, the human hairs in the scats probably came from deceased combatants on the battlefields and from corpses of civilians that were abandoned and left for hyenas. In Tigray, human bodies were lying mixed with domestic animals, many bodies remained unburied on the streets for days, because people were not allowed to bury the bodies of loved ones, and they ended up being eaten by hyenas (Chothia and Bekit 2022). Because coalition forces used human wave tactics (old fashioned warfare) to fight with the Tigray Defense Force (TDF), hundreds of thousands of combatants died and were consequently left for hyenas. Furthermore, family members were not allowed to bury the bodies of loved ones, and therefore the bodies of the deceased were abandoned and left for hyenas to scavenge on.

Our results are not surprising given the tactics employed during the war. Every year, hyenas remove at least 200 tons of potentially disease‐carrying livestock carcasses from Mekele, northern Ethiopia (Sonawane, Yirga, and Carter 2021). Hyenas in Tigray are providers of civic ecosystem services, known to remove organic waste and dead animals from the streets, thus removing pathogens that pose health risks to humans and livestock (Yirga et al. 2015; Sonawane, Yirga, and Carter 2021). The change in diet not only reflects an ecological change in the absolute and relative abundance of resources for hyenas in response to war but reflects a substantial alteration of their ecosystem service role.

### 6.2 Hyena Abundance in Battle and Control Sites

We also showed that the abundance of hyena varied between study sites and was significantly higher at battle sites compared to control sites. Hyenas in Ethiopia are typically adapted to living in urban areas and are specialized in feeding on organic waste (Yirga et al. 2015). They are opportunistic and can adapt to ecosystems by shifting from predatory to scavenging behavior when the opportunity occurs. A single hyena processes 983 kg of livestockcarcass a year (Sonawane, Yirga, and Carter 2021).

Our results align with the expectation that hyena abundance would increase in rural battle sites as a result of the changes in resource abundance, and increase livestock predation in those rural areas. The reported changes in hyena abundance and human−wildlife conflict were largely a function of reduced organic livestock waste availability in urban areas (people were starving), and increased availability of human carcasses in rural battle areas including around cemeteries. Hyenas may scavenge on human remains, becoming more habituated to areas of conflict.

### 6.3 Livestock Predation at Battle and Control Sites

We found that war‐driven changes in predator behavior increased reported livestock predation in rural areas defined as battlefield sites relative to urban areas. Our survey data indicated a strong association between scavenger abundance and increased reported livestock predation by hyenas, wolves, and leopards. Although this may be “coincidental” predation, armed conflict could also increase predation on livestock by limiting the family/farm management of domestic animals, unattended livestock during wartime becomes easy prey. Reported livestock depredation was significantly increased across the study sites during the war, which might be due to reduced availability of anthropogenic waste during the war (e.g., starvation was the norm). We previously reported that the reduced production of human organic waste during periods of religious fasting increases livestock predation in Tigray (Yirga et al. 2012). There is evidence that scavengers may turn to cattle predation during periods of reduced cattle waste production (Kalyahe, Hoferm, and East 2022).

Our research demonstrates the profound impact of war on predator diets and habitat use, driven by the availability of human remains—an often‐overlooked phenomenon. Wars disrupt ecosystems, altering resource availability and reshaping human−predator interaction in unpredictable ways. Armed conflict has been widely recognized as a significant driver of environmental and ecological change (Machlis and Hanson 2008). In our study, spotted hyenas' increased reliance on human remains and livestock during wartime exemplifies their ability to adapt to novel food resources. This aligns with the principles of the Optimal Foraging Theory (MacArthur and Pianka 1966), which posits that predators adjust their diets and behaviors to maximize energy gain under changing environmental conditions. Similar shifts in predator behavior have been documented in urban environments, where scavengers like spotted hyenas or coyotes exploit anthropogenic food sources (Bateman and Fleming, 2012).

Livestock predation data in this study are based on self‐reports from rural households, which may introduce bias or errors in predator identification. This study is also geographically specific, which may limit the generalizability of the findings to other conflict zones or ecosystems. Despite these limitations, the research provides valuable insights into the ecological impacts of war, particularly its effects on predator behavior, habitat use, and the dynamics of human−wildlife conflict.

### 6.4 A Global Perspective on War and Biodiversity

Across the globe, large carnivores are now expanding into rapidly growing human‐dominated areas (Chapron et al. 2014; Gompper, Belant, and Kays 2015), and they frequently alter their behavior in response to human influences (Haswell, Kusak, and Hayward 2017). Their adaptation to human areas can lead to increased conflicts, such as livestock predation or attacks on people.

Our results clearly showed a long‐term effect of war on hyena diets, hyena habitat use and regional abundance, and ultimately livestock predation rates. Our data specifically focus attention on the horrific potential for human remains and the impacts of war on managing the dead to drive regional‐scale changes in behavior and abundance of predators. We note that even after the war ended, the predation rates remain elevated, and distributions have not returned to pre‐war status where hyena abundance is centred in urban areas. War‐induced changes in foraging behavior of hyenas may possibly last longer. Recognizing that hyenas in urban areas traditionally also provide critical ecosystem services and health (Sonawane, Yirga, and Carter 2021; Yirga et al. 2024), there are implications of these changes for the return of civic order and for healthcare in the region.

However, it is worth noting that war does not always have negative effects on wildlife. Some wildlife populations benefit during wartime, for example, during Zimbabwe's civil war, elephant populations surged because the war kept poachers away from elephant habitats within the conflict zone (Rells 2019). Similarly, between North and South Korea in the Demilitarized Zone, wildlife populations are increasing due to the lack of anthropogenic disturbance (Rells 2019). Hyena populations certainly do not suffer in Tigray.

The war appears to have generated a major change in the human−wildlife interaction in Tigray: rural households in battle site areas experience higher abundance of hyenas, increased predation on livestock, and conflict than before the war reflecting a shift of hyena density from urban to rural areas and an associated loss of ecosystem service in the urban areas (Yirga et al. 2015; Yirga et al. 2024). Our work provides a crucial new perspective on the long‐term inhumane effects of armed conflict on civilians. These results yield insight not only into the consequences of war for the people of Tigray but also into how the many armed conflicts in regions with large scavenger/carnivore populations may have long‐lasting impacts on human−wildlife conflict around the globe. Through a comparison of battle sites to nonbattle sites, we demonstrate effects of war on the diet, abundance, distribution and hunting behavior of hyenas. The war has induced significant changes in the diet, abundance, distribution, and hunting behavior of spotted hyenas. By comparing battle sites to nonbattle sites, we demonstrate the long‐lasting ecological consequences of war. Future studies could explore how long these changes persist and their multigenerational effects on hyena populations.

## Author Contributions

**Gidey Yirga:** conceptualization (equal), writing – original draft (equal), writing – review and editing (equal). **Robert P. Freckleton:** writing – review and editing (equal). **Andrew P. Beckerman:** supervision (equal), writing – original draft (equal), writing – review and editing (equal).

## Conflicts of Interest

The authors declare no conflicts of interest.

## Data Availability

The authors confirm that the data supporting the findings of this study are available within the article.
